# Attitude towards diabetes mellitus among adult communities in Gondar city, Ethiopia

**DOI:** 10.1371/journal.pone.0251777

**Published:** 2021-05-20

**Authors:** Abiy Maru Alemayehu, Malede Mequanent Sisay

**Affiliations:** 1 Department of Optometry, School of Medicine, College of Medicine and Health Sciences, University of Gondar, Gondar, Ethiopia; 2 Department of Epidemiology and Biostatistics, Institute of Public Health, College of Medicine and Health Sciences, University of Gondar, Gondar, Ethiopia; University of the Free State, SOUTH AFRICA

## Abstract

**Background:**

Diabetes mellitus is a metabolic disorder characterized by hyperglycemia resulting from defects in insulin secretion, insulin action, or both. Diabetes and its complications can be reduced by enhancing the attitude of the community. However, there is limited information regarding attitude towards diabetes in northwest Ethiopia. Therefore, this study determined the attitude and associated factors of diabetes mellitus among adult non-diabetic participants in Gondar city.

**Methods:**

A community-based cross-sectional study was conducted in Gondar city. Systematic random sampling was employed to select 626 non-diabetic participants. The data were collected using a pre-tested structured questionnaire. Descriptive statistics, processing, and analysis were done using STATA version 14. Both bivariable and multivariable binary logistic regressions were used to identify the associated factors. An adjusted odds ratio with a 95% confidence interval was used to calculate a level of significance.

**Results:**

Of 626 participants, 572 (91.37%) study subjects heard about diabetes mellitus. Three hundred and fifteen participants (55.07%) (95% CI: 50.9% - 59.1%) had a favorable attitude towards diabetes mellitus. Having good knowledge about diabetes (adjusted odds ratio = 2.69, 95% CI: 1.88, 3.87), and higher educational status (adjusted odds ratio = 1.69, 95% CI: 1.04, 2.78) were positively associated with a favorable attitude towards diabetes mellitus. Female gender (adjusted odds ratio = 0.68, 95% CI: 0.47, 0.98), on the other hand, had poor attitude towards diabetes mellitus.

**Conclusion:**

In this study, a favorable attitude towards diabetes was low among adult non-diabetic participants. Good knowledge, higher educational status, and being male were the factors associated with a favorable attitude towards diabetes.

## Background

Diabetes mellitus (DM) is a metabolic disorder that results either from loss of insulin generating cells in the pancreas or reduced sensitivity of the tissues to insulin [1, 2]. It is one of the priority non-communicable diseases [3]. One-third of people with diabetes remain undiagnosed because of several factors [4]. If it is left undiagnosed and not treated, it causes blindness [3, 5, 6] and it affects other parts of the body [7–9].

Globally, the number of diabetic patients in 2019 was 463 million [10]. This number is estimated to be 552 million by the year 2030, suggesting a swift rise in DM prevalence [11]. Besides, the annual global diabetes health expenditure in 2019 was $760 billion [10].

The African region will be the epicenter of the epidemics in the next twenty-five years[10]. In Sub-Saharan Africa, DM is an important cause of morbidity and mortality [12, 13].

In Ethiopia, the national prevalence of DM was 3.2% in 2015 [14]. As revealed in the study done in Ethiopia, the number of patients is increasing from time to time [15]. It is also one of the prevalent non-communicable diseases in Ethiopia [16]. Besides, DM is one of the top leading causes of premature mortality and disability in Ethiopia [17].

Thus, attitude towards DM and its complications plays a fundamental role in the management of the disease and consequently, its burden. Previous studies in Ethiopia had revealed a significant attitude gap among the community towards DM and its complications [18, 19].

There is strong evidence that a lower level of knowledge is known to be associated with poor attitude as revealed in different studies [19–22]. Knowledge is one of the predictors of behavior in many ways [23–25]. The preliminary findings of our previous study showed that there was a low level of knowledge about DM. According to the findings, more than 58% of individuals with a poor level of knowledge had an unfavorable attitude towards DM. Besides, participants who had a low knowledge level regarding risk detection and the prevention methods to DM had an unfavorable attitude towards DM [26]. For the aforementioned reasons, we assessed and reported the cognitive aspects of DM in the community. An attitude drives our behavior that in turn brings true emotional insight. This indicates the need for studying the attitude of the community towards DM. The attitude of the participants can be improved by introducing a group-based collaborative strategy to prevent DM [27, 28].

Even though DM is one of Ethiopia’s rapidly rising public health problems, most studies focus on its prevalence and complications. The present study is intended to address these problems while serving as a baseline for further studies to generate inputs for health care providers and policymakers.

## Methods and material

### Study design, area, period, and population

We conducted a cross-sectional community-based study from August 1^st^ to August 25^th^ 2019 in Gondar city, northwest Ethiopia. The city had a population of 351, 675. It is divided into 10 sub-cities and 24 kebeles (the smallest administrative unit in Ethiopia) [29]. The city had various public and private health institutions. The public ones include a specialized referral university hospital and eight public health centers. The private sectors include nearly fifty clinics and one hospital. The included study participants were all adults older than 18 years old non-diabetic members who lived in Gondar city. However, participants with diabetes and those with other chronic systemic diseases were excluded.

### Sample size determination and sampling procedures

The sample size determination was made using the single population proportion formula with the following assumptions: a proportion of 55.9% [19], a 95% confidence interval, a 5% margin of error, 1.5 design effect, and 10% for non-response rate which gave a final sample size of 626. From a total of 24 kebeles, six kebeles were selected using a lottery method. The study participants were selected using a systematic random sampling technique from each household after getting a list of households from each kebele’s administration.

If a selected household was not accessible, the next household was included. When two or more participants were identified in the same household, one participant was chosen using the lottery method.

The University of Gondar ethical review board approved the research with a reference number V/P/RCS/05/2030/3019. Then, Gondar city administrative officials were communicated through formal letter. Permission to perform the study was taken from the head of the household. After explaining the purpose of the research, written informed consent was obtained from each study participant.

### Operational definitions

#### Awareness

If a positive response (‘Yes’) was obtained to the question ‘have you ever heard of diabetes mellitus?’

#### Non-diabetic community

A member of a community who has no known history of diabetes mellitus.

#### Attitude

The way a community thinks and behaves toward DM, measured by 10 items using a five-point Likert scale. The scoring system used for the participant’s responses was as follows: strongly agree 5, agree 4, neutral 3, disagree 2, and strongly disagree 1. The responses were summed up, and a total score was obtained for each respondent. The median was calculated, and those who scored above the median value (40) were considered to have favorable attitude, and those who scored less than the median value (40) had an unfavorable attitude regarding diabetes mellitus [18, 19].

#### Good knowledge

Individuals who responded mean (19.37) and above of the total knowledge questions were considered to have good knowledge about diabetes mellitus [26].

#### Poor knowledge

Individuals who responded below the mean (19.37) of the total knowledge questions were considered to have poor knowledge about diabetes mellitus [26].

### Data collection procedures and personnel

A pre-tested structured questionnaire was used to collect data after translating it into the Amharic version (the local language in Gondar city) from the English version and then back to English for consistency and ease of the interview. We reviewed different publications to develop the questionnaire [1, 18, 19, 21, 22, 30–36]. The questionnaire was organized to extract participants’ perceptions towards prevention methods, aggravating factors, and complications of DM. By taking 5% of the total sample size, a pretest was conducted in Bahirdar city. After the pretest, we assessed the face and content validity of the developed questions. The reliability of the questionnaire was checked with a Cronbach’s Alpha value of 0.779. Six trained optometrists took part in the data collection process.

### Data processing and analysis

After cleaning and coding, the data were entered into EpiData version 3.1. The analysis was done using STATA version 14. Proportions, rates, and summary statistics were calculated for most variables. We performed multivariable logistic regression to determine the associated factors. The variables with a P-value of less than 0.05 were considered statistically significant.

## Results

### Socio-demographic characteristics of respondents

Of the 626 respondents, 54 (8.63%) participants were not aware of DM. The analysis was done for the remaining 91.37% of participants who heard about diabetes mellitus.

Of the 572 respondents, 301 (52.62%) participants were female. About 332 (58.04%) respondents were married, and almost 80% of the study participants had secondary and above educational status.

The main sources of information were the mass media (42.83%). Of all study participants, 85.84% had a television or radio. Only 178 (31.12%) of the participants had exposure to DM health education (Table 1).

**Table 1:** Socio-demographic characteristics and source of information of respondents to assess attitude towards DM among adult non-diabetic participants in 2019 (n = 572).

Characteristics	Categories	Frequency	%
Sex	Female	301	52.62
	Male	271	47.38
Age in years	≤24	101	17.66
	25–34	212	37.06
	35–44	136	23.78
	≥ 45	123	21.50
Marital status	Single	204	35.66
	Married	332	58.04
	Divorced	21	3.67
	Widowed	15	2.62
Level of education	No formal education	97	16.96
	Primary school	34	5.94
	Secondary school & above	441	77.10
Occupation	Housewife	86	15.03
	Student	98	17.13
	Merchant	120	20.98
	Farmer	17	2.97
	Government/private worker	214	37.41
	Daily worker	19	3.32
	Others*	18	3.15
Family monthly income in Birr	≤ 2000	163	28.50
	2001–3000	123	22.03
	3001–5000	177	30.94
	≥ 5001	106	18.53
Level of knowledge	Poor	278	48.60
	Good	294	51.40
Awareness	Yes	572	91.37
	No	54	8.63
Exposure to DM health education	Yes	178	31.12
	No	394	68.88
Do you have a television?	Yes	491	85.84
	No	81	14.16
Family history of DM	Yes	79	13.81
	No	493	86.19
What is your source of information?	Mass Media	245	42.83
	Health personnel	124	21.68
	Colleague or relatives	177	30.94
	Others **	26	4.55

*Non-governmental workers, retired…

**teachers, pop…

### The attitude of participants towards diabetes mellitus

Almost one-third (35.84%) of the participants strongly agreed to the item “do you think that you should be examined for DM and your family members should be screened for DM”. While 43.45% of the participants strongly disagreed with the question “do you think controlling blood glucose level can prevent DM” (Table 2).

**Table 2:** Frequency distributions of respondents attitude towards diabetes mellitus among adult non-diabetes community members of Gondar city, Ethiopia 2019 (n = 572).

Question	Response option, n (%)				
	Strongly disagree	Disagree	Neutral	Agree	Strongly agree
I don’t mind if others know that I am with DM.	17(2.97)	59(10.31)	44(7.69)	281(49.13)	171(29.90)
Do you think that you should be examined for DM?	16(2.80)	31(5.42)	(38(6.64)	282(49.30)	205(35.84)
Do you think family members should be screened for DM?	9(1.57)	29(5.07)	35(6.12)	299(52.27)	200(34.97)
Do you think support from family and friends is important to control DM?	7(1.22)	18(3.15)	24(4.20)	254(44.41)	269(47.03)
Do you think should we follow avoiding of consumption of too much sugar for controlling DM?	17(2.97)	35(6.12)	62(10.84)	236(41.26)	222(38.81)
DM doesn’t seriously affecting the marital relationship.	37(6.47)	127(22.20)	124(21.68)	188(32.87)	96(16.78)
DM is not seriously affect the daily activities.	57(9.97)	161(28.15)	77(13.46)	188(32.87)	89(15.56)
Do you think physical activity can prevent the risk of DM?	9(1.57)	39(6.82)	31(5.42)	260(45.45)	233(40.73)
Do you think maintaining a healthy weight is important in the management of DM?	5(0.87)	44(7.69)	28(4.90)	250(43.71)	245(42.83)
DM complications may be prevented if blood glucose level is well controlled.	36(5.97)	23(3.81)	42(6.97)	240(39.80)	262(43.45)

**Fig 1:**
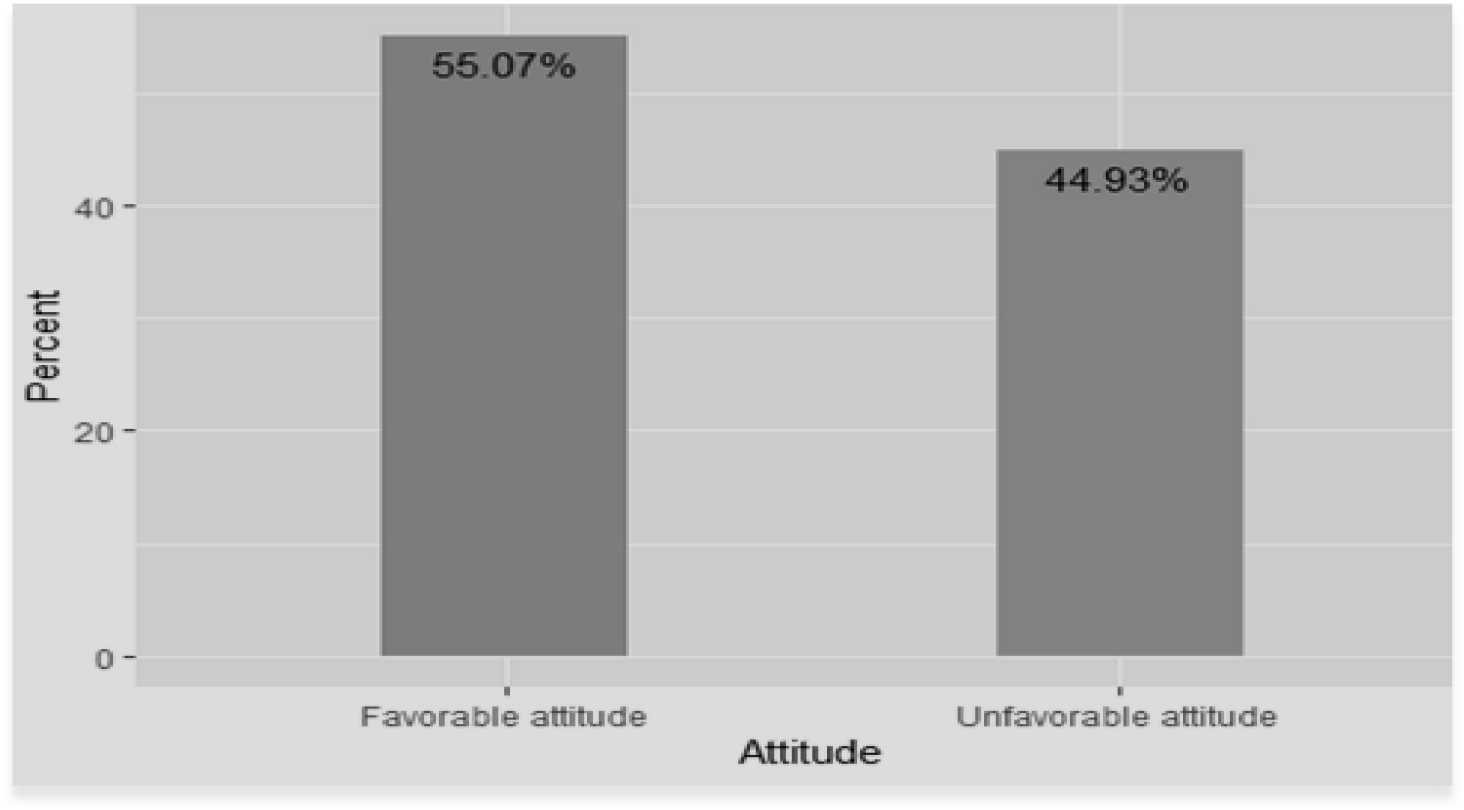
Level of attitude towards diabetes mellitus among adult non-diabetic community members of Gondar city, Ethiopia 2019.

The median score of the participant’s attitude was 40 (IQR: 37, 43). Three hundred and fifteen participants (55.07%) scored median and above (Fig 1).

### Factors associated with attitude level toward DM

In the multivariable logistic regression, female subjects were 32% (**AOR = 0.68, 95% CI: 0.47, 0.98**) less likely to have a favorable attitude towards diabetes as compared to males.

Individuals who had good knowledge about diabetes were 2.69 times (**AOR = 2.69, 95% CI: 1.88, 3.87**) more likely to have a favorable attitude towards diabetes as compared to those who did not have good knowledge.

Moreover, subjects who had higher educational levels were 1.69 times (**AOR = 1.69, 95% CI: 1.04, 2.78)** more likely to have a favorable attitude towards diabetes compared to those who did not have an education (Table 3).

**Table 3:** Bi- variable and multivariable logistic regression predicting DM related to attitude level among adult non-diabetic participants in Gondar city, Ethiopia 2019 (n = 572).

Variable	Category	Attitude level		COR (95% CI)	AOR (95%CI)
		Poor (%)	Good (%)		
**Marital status**	Married	152(59.19)	180(57.14)	1	1
	Unmarried*	105(40.86)	135(42.86)	1.09(0.78, 1.52)	1.14(0.79, 1.66)
**Sex**	Female	103(40.08)	168(53.33)	0.59(0.42, 0.82)	**0.68(0.47, 0.98)**
	Male	154(59.92)	147(46.67)	1	1
**Level of education**	No Education	61(23.74)	36(11.43)	1	1
	Primary	14(5.45)	20(6.35)	2.42(1.10, 5.37)	1.99(0.87, 4.56)
	Secondary and above	182(70.82)	259(82.22)	2.41(1.53, 3.79)	**1.69(1.04, 2.78)**
**Occupation**	Others**	95(36.96)	89(32.17)	0.67(0.47, 0.96)	0.73(0.49, 1.09)
	Working	162(63.04)	226(71.75)	1	1
**Family monthly income in Birr**	≤ 2000	77(29.96)	86(27.30)	1	1
	2001–3000	60(23.35)	66(20.95)	0.99(0.62, 1.57)	0.89(0.54, 1.46)
	3001–5000	74(28.79)	10.(32.70)	1.25(0.81, 1.91)	0.93(0.58, 1.49)
	≥ 5001	46(17.90)	60(19.05)	1.17(0.71, 1.91)	0.75(0.49, 1.30)
**Family history of DM**	No	225(87.55)	268(85.08)	1	1
	Yes	32(12.45)	47(14.92)	1.23(0.76, 1.99)	1.03(0.62, 1.71)
**Knowledge level**	Poor Knowledge	162(63.04)	116(36.83)	1	1
	Good Knowledge	95(36.96)	199(63.17)	2.93(2.08, 4.12)	**2.69(1.88, 3.87)**

*Single, Divorced/separated, Widowed.

** Housewives, Students.

## Discussion

In this study, about 8.6% of the study participants were not aware of diabetes mellitus. This shows there is a need for intervention in order to boost communities’ knowledge, which was low as indicated in our previous work [26]. It also had a relation with the attitude of individuals. Hence, those subjects who had poor knowledge had a poor attitude towards DM.

In this study, 315 (55.07%) non-diabetic adult community members had a favorable attitude regarding diabetes mellitus. This finding is in line with a study conducted in the Bale zone administrative town (55.9%) [19]. This is due to the expansion of health care services in both study areas through health extension programs [37] which can give favorable attitude towards DM. The other reason could be the similarity of the study design used in both studies.

However, this finding was higher than the study done in Debre Tabor (39.5%), Ethiopia [18]. The explanation for this is attributable to a difference in the scoring during the analysis and the number of items used to determine attitude. In this study, we used ten items with a maximum score of fifty, however, a different scoring was used in the study done in Debre Tabor. And also, it was higher than the study conducted in India (12.4%) [34]. This is because of socio-cultural differences between the two study populations. Besides, there is disparity in literacy level among study participants in both studies. In the study done in India 70.5% of the study participants had no formal education, whereas in this study 83.04% of study participants had primary and above educational status. Therefore, lower levels of educational status may lead to a poor attitude towards DM. Besides, this finding was higher than another study done in India (17.58%) [38]. This is due to the difference in the number of items and the measurement method used. The study done in India used only five items and coded the scoring in a ‘yes’ or ‘no’ format, which might not measure the attitude level well. The finding in this study was also higher than studies done in Kenya (49%) [33], Saudi Arabia (48%) [22], and Sri Lanka (10%) [35]. This may be due to the sample size difference, which was higher in this study. Also, the level of attitude was higher in this study when compared to the study done in Jordan (46.3%) [36]. The likely reason is the population difference in the two studies. The study done in Jordan included populations from different geographical locations, making variation higher. Hence, the measure might not be an accurate reflection of the population’s attitude in Jordanian study population.

In this study, being male, educational level, and knowledge level towards DM were significantly associated with the attitude toward DM.

The odds of a favorable attitude towards DM among study participants with secondary school and higher educational qualification were 1.66 times greater than compared to study subjects with no formal education. This finding was in line with studies done in Ethiopia [18, 39], Jordan [36], India [34], and Kenya [33]. Because, a higher level of educational status can bring behavioral change among individuals, thereby increasing their attitude. On the other hand, as individuals learn more, the chance of gaining information about DM from different sources will increase. Also, those individuals with a high level of education can read and understand different medical information. Therefore, these can give a favorable attitude. However, in the study done in Sri Lanka, the level of education was not associated with attitude towards DM [35]. This may be due to the larger sample size used in this study than the study done in Sri Lanka.

The odds of a favorable attitude towards diabetes mellitus among study participants who had good knowledge about DM were 2.67 times greater than the odds of a favorable attitude of study subjects who had poor knowledge about DM. This finding is consistent with studies done in the Bale administrative zone, Ethiopia [19], South Africa [20], Bangladesh [21], and Saudi Arabia [22]. This may be because knowledgeable individuals have an opportunity to interact with other people and share ideas and feelings. Furthermore, such feelings might contribute to the development of a favorable attitude towards DM. It is clear that as knowledge increases, individuals will have a tendency to attend health education campaigns or watch any media like television, which is one means of gaining information.

However, females were 32% less likely to have a favorable attitude towards diabetes as compared to males. This might be due to less access to mass media among females as compared to males [40]. Access to information has a significant advantage by enhancing one’s attitude, so less exposure to information among females might lead to a less favorable attitude towards the disease. The other possible reason is a cultural influence that pushes females to spend time in the home activities, but males spend most of their time outside home [41]. This may give men more opportunities to acquire more information and to attend different meetings and conferences.

## Limitation of the study

Since it was a cross-sectional study, it did not show the conditions of cause and effect relationships. Also, it is difficult to generalize for the whole population since the study excluded homeless, diabetic patients, patients with chronic disease, and street dwellers.

## Conclusion

In this study, favorable attitude towards diabetes mellitus was low among adult non-diabetic community in Gondar city.

Good knowledge about diabetes mellitus, a high level of educational status, and being male were the factors associated with a favorable attitude of participants towards diabetes mellitus. To improve the attitude towards diabetes mellitus, it is necessary to strengthen community-based health education programs. Females need to be given greater emphasis. Further studies with a solid study design and other important variables like sociocultural and behavioral components need to be considered.

## Supporting information

S1 Data Data set. (DTA)
